# Factors Associated With Weight Change After Continuing or Switching to a Doravirine-based Regimen

**DOI:** 10.1093/ofid/ofaf639

**Published:** 2025-11-20

**Authors:** Chloe Orkin, John R Koethe, Princy N Kumar, Peter Sklar, Zhi Jin Xu, Rebeca M Plank, Wayne Greaves, Rima Lahoulou

**Affiliations:** SHARE Collaborative, Blizard Institute, Queen Mary University of London, London, UK; Division of Infectious Diseases, Vanderbilt University Medical Center, Nashville, Tennessee, USA; Division of Infectious Diseases and Tropical Medicine, Medstar Georgetown University Hospital, Washington, DC, USA; MRL Research Laboratories, Merck & Co., Inc., Rahway, New Jersey, USA; MRL Research Laboratories, Merck & Co., Inc., Rahway, New Jersey, USA; MRL Research Laboratories, Merck & Co., Inc., Rahway, New Jersey, USA; MRL Research Laboratories, Merck & Co., Inc., Rahway, New Jersey, USA; MRL Research Laboratories, Merck & Co., Inc., Rahway, New Jersey, USA

**Keywords:** demographics, doravirine, HIV-1, switch, weight

## Abstract

**Background:**

Factors associated with weight change were examined in phase 3 studies in which adults living with HIV-1 continued or switched to doravirine-based antiretroviral regimens.

**Methods:**

Participants were randomized to first-line therapy with doravirine or darunavir/ritonavir, each given with 2 nucleos(t)ide reverse transcriptase inhibitors (NRTIs) (DRIVE-FORWARD) and to doravirine/lamivudine/tenofovir disoproxil fumarate (TDF) or efavirenz/emtricitabine/TDF (DRIVE-AHEAD); after 96 weeks, participants continued (n = 466) or switched to (n = 423) doravirine for 96-week open-label extensions. In DRIVE-SHIFT, virologically suppressed participants on stable antiretroviral therapy were randomized to switch to doravirine/lamivudine/TDF at day 1 or week 24 through week 144 (n = 535). Generalized logistic models were used to analyze factors associated with weight loss (≥5% decrease), stable weight (<5% change), and weight gain (≥5% increase) after continuation or switch.

**Results:**

Most participants who continued or switched to doravirine also received TDF (>74%) and had stable weight (>57%). Weight loss (odds ratio [OR], 6.14) or stable weight (OR, 2.15) was more common in participants of non-Black versus Black heritage after switching to doravirine. More participants switching from weight-suppressive non-nucleoside reverse transcriptase inhibitors versus protease inhibitors experienced weight gain versus weight loss in DRIVE-SHIFT (OR, 0.41) and weight gain versus stable weight in DRIVE-FORWARD and DRIVE-AHEAD (OR, 0.60). No significant association between weight change and prior NRTI use was observed in DRIVE-SHIFT.

**Conclusions:**

Overall, switching to doravirine was weight neutral, although weight change may differ by demographics and weight-suppressive properties of a prior regimen. More research in historically underrepresented groups may help explain these findings.

**ClinicalTrials.gov:**

NCT02275780, NCT02403674, NCT02397096.

## Teaser

In three phase 3 clinical studies, switching to a doravirine-based regimen was weight neutral in most adults with HIV-1, although weight change differed across demographic subgroups and according to weight-suppressive properties of a prior regimen.

Antiretroviral therapy (ART) has extended the life expectancy of people living with HIV-1; in a recent analysis of North American and European cohorts, life expectancy of those on long-term ART is similar to that of the general population [1]. While advances in HIV treatment have prolonged lives, this success has been accompanied by a rise in comorbid conditions, such as hypertension, diabetes mellitus, weight gain, and obesity, across diverse groups and regions [2]. Older age and higher body weight contribute to the risk of comorbid conditions, and the proportion of people living with HIV who are overweight or obese has been increasing globally and is now >50% in the United States [3, 4]. While some weight gain after initiating ART is considered a return-to-health phenomenon, excessive weight gain over time increases inflammation risks such as cardiovascular disease, metabolic syndrome, mobility disorders, and diabetes mellitus [3–6].

Debates about weight gain in the context of HIV and ART have centered on whether ART agents have independent effects on body weight above and beyond the return-to-health phenomenon accompanying the reversal of HIV-induced catabolism and muscle wasting [3]. Furthermore, certain demographic and immunologic baseline parameters have been associated with weight gain (eg, sex, Black heritage, and lower CD4 count) [3, 7, 8]. Clarifying this issue is of major clinical importance, as even moderate weight gain on ART confers a higher risk of developing metabolic syndrome in those living with HIV compared with those without HIV. Approximately 1 in 5 people living with HIV move into a higher weight category within 3 years of initiating ART (from normal to overweight or overweight to obese) [3, 8]. Furthermore, much of this weight accumulates in the viscera, liver, skeletal muscle, and other organs, which contributes to an increased risk of physical frailty, liver disease, and cognitive decline in those living with HIV over the age of 50 years [9].

Several studies have shown that treatment with integrase strand transfer inhibitors (InSTIs) and tenofovir alafenamide (TAF), compared with other classes of ART, is associated with increased weight gain [9–17]. This weight gain was observed after switching to newer regimens containing InSTIs and/or TAF and is hypothesized to reflect the loss of a weight-attenuating effect of older drugs contained in previous regimens such as tenofovir disoproxil fumarate (TDF) or efavirenz (EFV) [18]. Most recent clinical studies support weight neutrality for the InSTIs dolutegravir, cabotegravir, and bictegravir, as well as the nucleos(t)ide reverse transcriptase inhibitor (NRTI) TAF [19]. Participants receiving TDF, especially when combined with EFV, show weight attenuation in clinical studies of antiretrovirals as pre-exposure prophylaxis, first-line treatment, and switch therapy [19].

Minimal weight gain was observed with doravirine (DOR)-based regimens as first-line therapy in the DRIVE-FORWARD (DOR + 3TC + TDF or ABC) and DRIVE-AHEAD (DOR/3TC/TDF) studies: median weight gain over a period of up to 4 years of treatment was approximately 2 kg in both studies [20], similar to the average yearly increase observed among US adults without HIV [21]. Likewise, participants switching from a stable ART regimen to DOR with lamivudine (3TC) and TDF in the DRIVE-SHIFT study had a mean weight gain of 1.2–1.4 kg from the time of switch (day 1 or week 24) to week 144 [22].

In the three phase 3 clinical studies of DOR-based regimens mentioned above, most participants (∼70%) experienced <5% weight gain [22, 23]. Observational cohort data also suggest that DOR treatment is associated with minimal weight gain [24, 25]. Two studies evaluating a switch to DOR/islatravir (ISL, a novel nucleoside reverse transcriptase translocation inhibitor) from either bictegravir/emtricitabine (FTC)/TAF or any baseline ART showed weight effects after switching to DOR/ISL was related to the previous ART regimen [26, 27]. The pooled analysis presented here aimed to characterize participants who maintained weight or experienced weight change after continuing or switching to a DOR-based regimen in the DRIVE-FORWARD, DRIVE-AHEAD, and DRIVE-SHIFT phase 3 clinical studies.

## METHODS

### Study Design

DRIVE-FORWARD (MK-1439-018; NCT02275780) and DRIVE-AHEAD (MK-1439A-021; NCT02403674) were randomized, double-blind, active-controlled, noninferiority studies of first-line ART in adults living with HIV; both studies included a 96-week base study and a 96-week open-label extension study (Supplementary Figure 1). For DRIVE-FORWARD, participants were randomly assigned (1:1) to DOR 100 mg or darunavir 800 mg with ritonavir 100 mg (DRV/r), each administered once daily with 2 NRTIs, either FTC 200 mg with TDF 300 mg (FTC/TDF; 87%) or abacavir 600 mg with 3TC 300 mg (ABC/3TC; 13%) [28]. In a study extension, participants continued or switched to DOR plus 2 NRTIs at week 96; changes in NRTIs between the base study and the study extension were made on a case-by-case basis, as needed, by agreement of the investigator and the sponsor, and TAF was permitted. For DRIVE-AHEAD, participants were randomly assigned (1:1) to DOR/3TC/TDF (100/300/300 mg) or EFV/FTC/TDF (600/200/300 mg). In a study extension, participants continued or switched to DOR/3TC/TDF at week 96. Participants who continued or switched to DOR in the study extension for both DRIVE-FORWARD and DRIVE-AHEAD were pooled for analysis. For the pooled DOR continued group from DRIVE-FORWARD and DRIVE-AHEAD, 434 (93.1%) of 466 participants had TDF in their regimen. For the pooled DOR switch group, 399 (94.3%) of 423 participants had TDF in their regimen.

DRIVE-SHIFT was a randomized, open-label, active-controlled, noninferiority study of adults who were virologically suppressed and on stable ART for at least 6 months before enrollment (Supplementary Figure 2). Participants were randomly assigned (2:1) to switch to DOR/3TC/TDF on day 1 (immediate switch group) or to continue their baseline ART and switch to DOR/3TC/TDF at week 24 (delayed switch group). At week 48, eligible participants could enter the study extension and continue receiving open-label DOR/3TC/TDF for an additional 96 weeks (up to week 144). For the pooled switch group from DRIVE-SHIFT, 398 (74.4%) of 535 participants had TDF in their prior regimen.

### Study Population

Key eligibility criteria for the DRIVE-FORWARD, DRIVE-AHEAD, and DRIVE-SHIFT studies have been described previously [29–31]. All studies were performed in accordance with the International Conference on Harmonisation Good Clinical Practice guidelines and the principles of the Declaration of Helsinki. Institutional review board approval was obtained at each participating center. All participants provided written informed consent before enrollment.

### Statistical Analyses

For DRIVE-FORWARD and DRIVE-AHEAD, percentage weight change was calculated based on the difference between week 96 and week 192. For DRIVE-SHIFT, percentage weight change was calculated based on the difference between week 24 and week 144. Only participants with weight measurements at both time points were included in the analysis. Percent weight change was categorized as weight loss (≥5% decrease), stable weight (<5% change), or weight gain (≥5% increase).

Odds ratios, 95% CIs, and *P*-values for weight loss or stable weight versus weight gain were obtained from a generalized logistic model, with the status of weight change (loss, stable, and gain) as the outcome variable and the following exploratory variables. For the pooled DOR continued and DOR switch populations from DRIVE-FORWARD and DRIVE-AHEAD, exploratory variables included prior ART (protease inhibitor [PI], non-nucleoside reverse transcriptase inhibitor [NNRTI]), sex, ethnicity (Black heritage, non-Black heritage), day 1 age (<50 years, ≥50 years), week 96 body mass index (BMI) group (underweight/normal [<25 kg/m^2^], overweight [25–30 kg/m^2^], and obese [>30 kg/m^2^]), and week 96 weight. Interaction of sex and ethnicity was not included because there were no females of Black heritage in the weight loss group. For the pooled switch population from DRIVE-SHIFT (immediate and delayed switch cohorts), exploratory variables were sex, ethnicity (Black heritage, non-Black heritage), interaction of sex and ethnicity, day 1 age (<50 years, ≥50 years), week 24 BMI group (underweight/normal, overweight, obese), prior ART (PI, NNRTI, InSTI), NRTI in prior regimen (TAF, TDF, ABC, other), duration of prior ART (<1 year, ≥1 year), and week 24 weight. At the time of switch to or continuation of DOR across the 3 studies, most participants were virologically suppressed (HIV-1 RNA ≤50 copies/mL), and median CD4 counts were >600 cells/mm^3^; therefore, these variables were not included in the generalized logistic model.

## RESULTS

The pooled DOR continued population from DRIVE-FORWARD and DRIVE-AHEAD comprised 466 participants (434 [93.1%] received TDF): 55 (11.8%) lost weight, 305 (65.5%) had stable weight, and 106 (22.7%) gained weight (Table 1). Most participants were White males with a median age ranging from 33.0 to 34.0 years, and the mean weight ranged from 76.0 to 79.8 kg across weight change categories at week 96 (Table 1). Participant characteristics were generally similar between the weight change categories, except that the weight loss group had a numerically higher proportion of female participants. For the DOR continued population, no clinical or demographic factors were associated with weight loss or stable weight versus weight gain from weeks 96 to 192 (Supplementary Figure 3).

**Table 1.** Participant Characteristics by Weight Change Category in the DRIVE-FORWARD and DRIVE-AHEAD Studies

	DOR Continued Group, *N* = 466			DOR Switch Group, *N* = 423		
	Lost weight (≥5% decrease), *n* = 55 (11.8)	Stable weight (<5% change), *n* = 305 (65.5)	Gained weight (≥5% increase), *n* = 106 (22.7)	Lost weight (≥5% decrease), *n* = 40 (9.5)	Stable weight (<5% change), *n* = 243 (57.4)	Gained weight (≥5% increase), *n* = 140 (33.1)
Age, median (range), y	34.0 (18–63)	33.0 (18–70)	33.0 (18–68)	29.0 (19–56)	33.0 (19–67)	30.5 (18–69)
Sex						
Male	40 (72.7)	265 (86.9)	83 (78.3)	33 (82.5)	211 (86.8)	121 (86.4)
Female^a,b^	15 (27.3)	40 (13.1)	23 (21.7)	7 (17.5)	32 (13.2)	19 (13.6)
Ethnicity						
American Indian or Alaska Native	0	5 (1.6)	1 (0.9)	0	4 (1.6)	0
Asian	4 (7.3)	39 (12.8)	10 (9.4)	7 (17.5)	22 (9.1)	22 (15.7)
Black heritage^a,b^	10 (18.2)	44 (14.4)	22 (20.8)	3 (7.5)	32 (13.2)	31 (22.1)
Multiple	5 (9.1)	30 (9.8)	5 (4.7)	3 (7.5)	23 (9.5)	12 (8.6)
Native Hawaiian/other Pacific Islander	NA	NA	NA	0	1 (0.4)	1 (0.7)
White	36 (65.5)	187 (61.3)	68 (64.2)	27 (67.5)	161 (66.3)	74 (52.9)
TDF in regimen	52 (94.5)	283 (92.8)	99 (93.4)	35 (87.5)	230 (94.7)	134 (95.7)
Week 96 weight, kg						
Mean (SD)	79.8 (15.8)	76.2 (16.1)	76.0 (15.5)	79.8 (14.0)	75.2 (15.5)	74.0 (15.6)
Median (range)	77.1 (52.0–136.1)	74.0 (41.4–135.0)	73.7 (48.0–134.5)	75.0 (60.0–112.5)	74.0 (42.7–139.3)	72.7 (38.0–124.3)
Week 96 BMI, kg/m^2^						
Mean (SD)	27.0 (5.3)	25.4 (5.1)	25.4 (5.1)	25.9 (3.6)	24.9 (4.6)	24.6 (4.7)
Median (range)	26.2 (16.7–43.0)	24.6 (16.4–56.4)	24.7 (16.0–49.7)	25.4 (20.2–38.8)	24.4 (14.9–48.0)	24.6 (16.6–44.6)
Week 96 BMI group						
Underweight	2 (3.6)	9 (3.0)	7 (6.6)	0	12 (4.9)	10 (7.1)
Normal	23 (41.8)	155 (50.8)	52 (49.1)	18 (45.0)	124 (51.0)	69 (49.3)
Overweight	15 (27.3)	97 (31.8)	30 (28.3)	19 (47.5)	76 (31.3)	46 (32.9)
Obese	15 (27.3)	43 (14.1)	17 (16.0)	3 (7.5)	30 (12.3)	15 (10.7)
Missing	0	1 (0.3)	0	0	1 (0.4)	0

Data are expressed as n (%) unless otherwise noted.

Abbreviations: BMI, body mass index; DOR, doravirine; TDF, tenofovir disoproxil fumarate.

^a^At week 96 for the DOR continued group (*N* = 466), the percentages of female, Black heritage, and female participants of Black heritage were 16.9%, 18.2%, and 7.6%, respectively.

^b^At week 96 for the DOR switch group (*N* = 423), the percentages of female, Black heritage, and female participants of Black heritage were 13.5%, 17.1%, and 5.8%, respectively.

**Figure 1.**
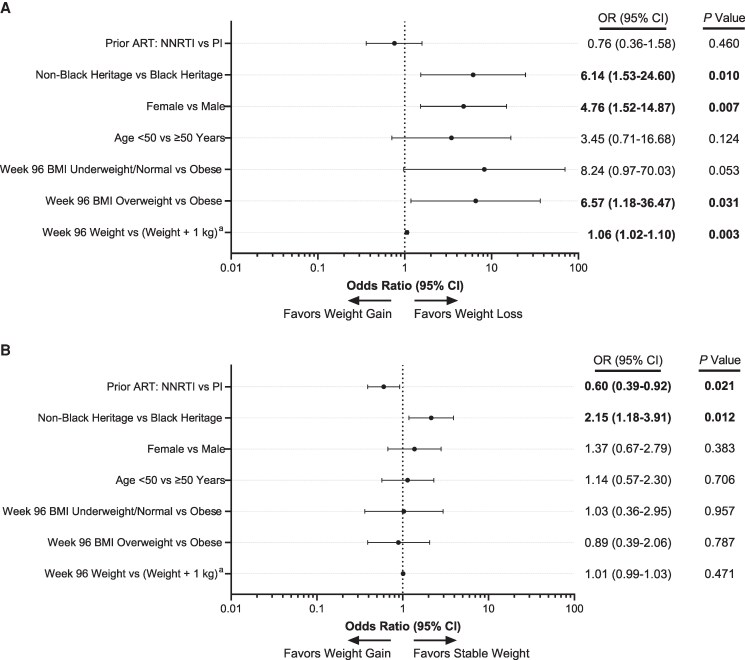
Analysis of factors impacting the probability of (*A*) weight loss or (*B*) stable weight versus weight gain from week 96 to week 192, DOR switch group, DRIVE-FORWARD and DRIVE-AHEAD. Bold denotes statistically significant variables (*P* < .05). ART, antiretroviral therapy; BMI, body mass index; DOR, doravirine; NNRTI, non-nucleoside reverse transcriptase inhibitor; OR, odds ratio; PI, protease inhibitor. ^a^For each additional kg of weight, the odds ratio for week 96 weight versus (weight + 1 kg) represents the ratio of the odds of two groups, one with weight X and another with weight (X + 1).

The pooled DOR switch population from DRIVE-FORWARD and DRIVE-AHEAD comprised 423 participants (399 [94.3%] received TDF): 40 (9.5%) lost weight, 243 (57.4%) had stable weight, and 140 (33.1%) gained weight (Table 1). Most participants included in the studies were White males with a median age ranging from 29.0 to 33.0 years, and the mean weight ranged from 74.0 to 79.8 kg across weight change categories at week 96 (Table 1). The weight loss group had the lowest proportion of participants of Black heritage: 3 (7.5%) compared with 32 (13.2%) in the stable weight group and 31 (22.1%) in the weight gain group. After switching to DOR, weight loss was more likely to occur in participants of non-Black heritage than in participants of Black heritage (*P* = .010), in females than in males (*P* = .007), and in overweight than in obese participants (*P* = .031; Figure 1*A*). Stable weight was more likely to occur in participants of non-Black heritage than in participants of Black heritage (*P* = .012), and after switch from DRV/r (prior ART: PI) than after switch from EFV/FTC/TDF (prior ART: NNRTI) (*P* = .021; Figure 1*B*).

**Figure 2.**
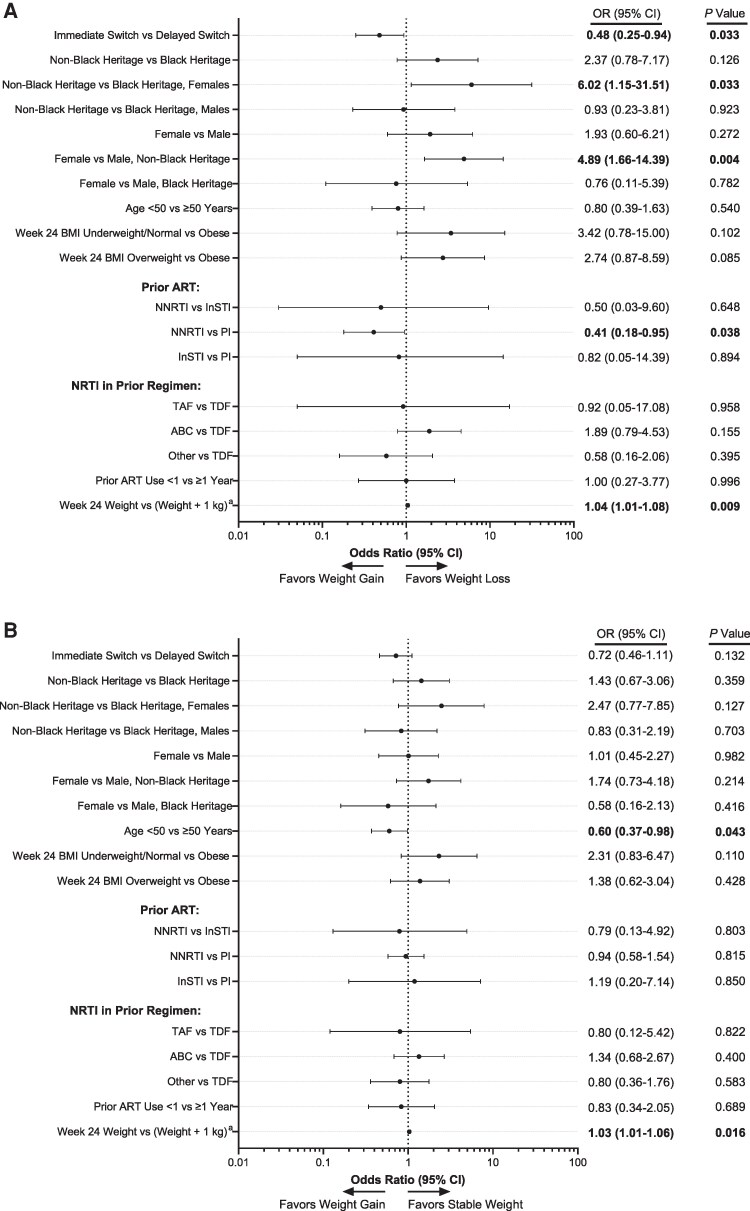
Analysis of factors impacting the probability of (*A*) weight loss or (*B*) stable weight versus weight gain from week 24 to week 144 in the DRIVE-SHIFT study. Bold denotes statistically significant variables (*P* < .05). ABC, abacavir; ART, antiretroviral therapy; BMI, body mass index; InSTI, integrase strand transfer inhibitor; NNRTI, non-nucleoside reverse transcriptase inhibitor; NRTI, nucleos(t)ide reverse transcriptase inhibitor; OR, odds ratio; PI, protease inhibitor; TAF, tenofovir alafenamide; TDF, tenofovir disoproxil fumarate. ^a^For each additional kilogram of weight, the odds ratio for week 24 weight versus (weight + 1 kg) represents the ratio of the odds of two groups, one with weight X and another with weight (X + 1).

The pooled switch population from DRIVE-SHIFT (immediate and delayed switch cohorts) comprised 535 participants (398 [74.4%] received TDF in their prior regimen): 71 (13.3%) lost weight, 340 (63.6%) had stable weight, and 124 (23.2%) gained weight (Table 2). Most participants were White males with a median age ranging from 41.0 to 44.0 years, and the mean weight ranged from 76.3 to 81.0 kg across weight change categories at week 24 (Table 2). The weight loss group had a numerically higher proportion of females (16 [22.5%]) and participants with prior PI use (59 [83.1%]) than the stable weight (47 [13.8%] and 235 [69.1%], respectively) and weight gain (18 [14.5%] and 84 [67.7%], respectively) groups. The weight loss group had a numerically lower proportion of participants with prior NNRTI use (9 [12.7%]) than the stable weight (86 [25.3%]) and weight gain (33 [26.6%]) groups. In DRIVE-SHIFT, participants who switched to DOR/3TC/TDF at day 1 were more likely to have weight loss at week 144 than those who switched to DOR/3TC/TDF at week 24 (*P* = .033; Figure 2*A*). Females of non-Black heritage were more likely to have weight loss than males of non-Black heritage (*P* = .004) or females of Black heritage (*P* = .033), after switching to DOR/3TC/TDF (Figure 2*A*). Weight loss was also more likely to occur in participants who switched from PIs than in those who switched from NNRTIs (*P* = .038; Figure 2*A*). There was no significant association with weight change and NRTI in the prior regimen (Figure 2*B*). Stable weight was more likely to occur in participants aged ≥50 years than in those <50 years (*P* = .043; Figure 2*B*).

**Table 2.** Participant Characteristics by Weight Change Category in the DRIVE-SHIFT Study

	Lost Weight (≥5% Decrease), *n* = 71 (13.3)	Stable Weight (<5% Change), *n* = 340 (63.6)	Gained Weight (≥5% Increase), *n* = 124 (23.2)
Age, median (range), y	44.0 (21–66)	44.0 (22–71)	41.0 (25–66)
Sex			
Male	55 (77.5)	293 (86.2)	106 (85.5)
Female	16 (22.5)	47 (13.8)	18 (14.5)
Ethnicity			
American Indian or Alaska Native	1 (1.4)	3 (0.9)	3 (2.4)
Asian	2 (2.8)	13 (3.8)	4 (3.2)
Black heritage^a^	7 (9.9)	36 (10.6)	15 (12.1)
Multiple	6 (8.5)	16 (4.7)	11 (8.9)
Native Hawaiian/other Pacific Islander	1 (1.4)	0	0
White	54 (76.1)	272 (80.0)	91 (73.4)
Prior ART			
PI	59 (83.1)	235 (69.1)	84 (67.7)
NNRTI	9 (12.7)	86 (25.3)	33 (26.6)
InSTI	3 (4.2)	19 (5.6)	7 (5.6)
NRTI in prior regimen			
TAF	3 (4.2)	17 (5.0)	6 (4.8)
TDF	50 (70.4)	255 (75.0)	93 (75.0)
ABC	14 (19.7)	45 (13.2)	13 (10.5)
Other	4 (5.6)	23 (6.8)	12 (9.7)
Week 24 weight, kg			
Mean (SD)	81.0 (19.1)	79.9 (17.1)	76.3 (13.2)
Median (range)	78.1 (53.0–196.6)	78.0 (39.8–150.7)	76.2 (40.0–122.0)
Week 24 BMI, kg/m^2^			
Mean (SD)	26.8 (5.1)	26.1 (5.2)	25.2 (4.0)
Median (range)	26.0 (19.0–50.1)	25.1 (16.4–54.4)	24.8 (14.2–36.6)
Week 24 BMI group			
Underweight	0	6 (1.8)	2 (1.6)
Normal	29 (40.8)	162 (47.6)	63 (50.8)
Overweight	30 (42.3)	111 (32.6)	42 (33.9)
Obese	12 (16.9)	60 (17.6)	17 (13.7)

Data are expressed as n (%) unless otherwise noted.

Abbreviations: ABC, abacavir; ART, antiretroviral therapy; BMI, body mass index; InSTI, integrase strand transfer inhibitor; NNRTI, non-nucleoside reverse transcriptase inhibitor; NRTI, nucleos(t)ide reverse transcriptase inhibitor; PI, protease inhibitor; TAF, tenofovir alafenamide; TDF, tenofovir disoproxil fumarate.

^a^At week 24 the percentages of female participants, participants of Black heritage, and female participants of Black heritage were 15.5%, 13.4%, and 6.0%, respectively.

## DISCUSSION

Stable weight (<5% change) was observed in most participants who continued DOR or switched to DOR in the DRIVE-FORWARD, DRIVE-AHEAD, and DRIVE-SHIFT clinical studies. Most participants who received DOR also received TDF + 3TC. No clinical or demographic factors were consistently identified as being associated with weight change in participants who continued their DOR regimen in the extension phase in DRIVE-FORWARD and DRIVE-AHEAD.

Among virologically suppressed participants who switched to DOR in the extension phase of the DRIVE-FORWARD and DRIVE-AHEAD studies, weight loss was significantly associated with female sex, non-Black heritage, and lower BMI in overweight versus obese categories. Among virologically suppressed participants who switched to DOR in the DRIVE-SHIFT study, weight loss was significantly associated with females of non-Black heritage, immediate switch from baseline ART, and switch from boosted PI. In DRIVE-FORWARD and DRIVE-AHEAD, stable weight was significantly associated with non-Black heritage and switch from boosted PI, whereas in DRIVE-SHIFT, older age (≥50 years) was significantly associated with stable weight. These results are consistent with earlier reports that participants of non-Black heritage were more likely to experience weight loss than participants of Black heritage [10, 15]. It has been speculated that socioeconomic advantages and an increased prevalence of ART-related gastrointestinal adverse events (loss of appetite, diarrhea, vomiting) among persons of non-Black versus Black heritage living with HIV could account for some of the ethnicity-related differences in ART-related weight gain [7]. There is limited evidence to date on the effects of switching ART on weight [3, 9, 32], highlighting the importance of showing that switching to a DOR + TDF–containing regimen is associated with weight neutrality for most people. Switching ART is not thought to result in reduced visceral adipose tissue [9] and is not recommended for people with weight gain alone [32].

TDF is an NRTI that has previously shown weight attenuation effects in those with and without HIV [17, 33–37]. In DRIVE-SHIFT, 398 of 535 participants (74.4%) had TDF as part of their prior regimen. Although no significant association between weight change and the type of NRTI in the prior regimen was observed after switch to a DOR-based regimen in this study, the sample size is relatively small. Further research is needed to clarify the singular effects of DOR on weight with and without the coadministration of TDF.

When assessing weight gain, differences based on demographic subgroups need to be considered. Every kilogram gained contributes to an increase in BMI and the possibility of moving from the overweight to obese threshold. However, whether these relatively greater increases in weight among females and in Black heritage groups are related to increased lean versus fat gains needs to be clarified. Also, when fat is gained the relative metabolic risk differs between subcutaneous and visceral adipose tissue.

Evidence is needed to help elucidate the specific impacts of ART-related weight gain on clinical outcomes and adverse events and to show if these adverse events differ from those seen with weight gain in the general population not living with HIV [6, 9, 38]. Accordingly, there is an unmet need for novel ARTs that offer comparable efficacy with a reduced risk of weight gain or other metabolic disorders, pending a greater understanding of the full implications of treatment-related weight gain for people living with HIV.

Due to the low numbers of female participants and participants of Black heritage in the DRIVE clinical study program, groups that have been underrepresented in clinical studies in general, more intentionally inclusive research is needed to better characterize weight gain and clinical outcomes in these groups. Additional follow-up is also required to further understand the long-term relationship between weight gain and metabolic outcomes and clinical events in people living with HIV. The weight-attenuating effect of TDF may be different for individuals who have been receiving ART for 4–7 years compared with those, such as the participants in DRIVE-FORWARD and DRIVE-AHEAD, who had only received ∼2 years of treatment before continuing or switching to a DOR-based regimen in the extension phase. Currently unidentified genetic polymorphisms may impact antiretroviral concentrations, which could impact changes in weight during treatment (eg, loss-of-function polymorphisms such as CYP2B6 result in higher EFV concentrations [39], which we hypothesized could impair weight gain among people living with HIV). However, no genetic data were collected in these studies.

## CONCLUSION

Overall, switching to a DOR-based regimen was weight neutral for participants in three phase 3 studies, with certain demographic subgroups and prior regimens associated with weight change. More research in historically underrepresented groups may help clarify these findings.

## Supplementary Material

ofaf639_Supplementary_Data

## Supplementary Data

Supplementary materials are available at *Open Forum Infectious Diseases* online. Consisting of data provided by the authors to benefit the reader, the posted materials are not copyedited and are the sole responsibility of the authors, so questions or comments should be addressed to the corresponding author.
