# Supplementary material for: Factors Associated With Weight Change After Continuing or Switching to a Doravirine-based Regimen
Source: Open Forum Infect Dis. 2025 Nov 20;12(11):ofaf639. doi: 10.1093/ofid/ofaf639 (PMC12631767; doi:10.1093/ofid/ofaf639)
Supplement: ofaf639_Supplementary_Data [file ofaf639_supplementary_data.docx]

**Supplementary Materials**

**Supplementary Figure 1. Study design of DRIVE-FORWARD and DRIVE-AHEAD**

**
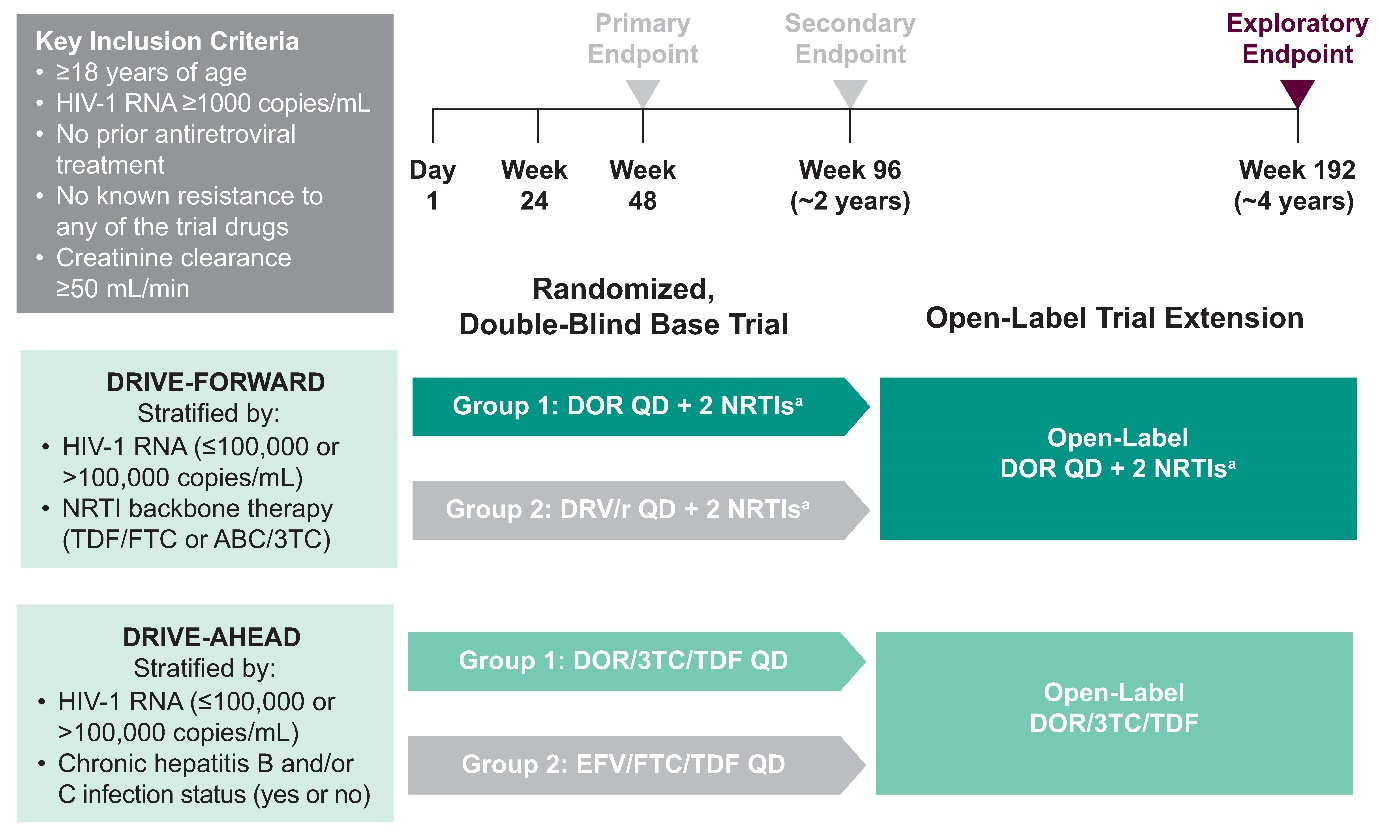
**

3TC, lamivudine; ABC, abacavir; DOR, doravirine; DRV/r, ritonavir-boosted darunavir; EFV, efavirenz; FTC, emtricitabine; NRTI, nucleos(t)ide reverse transcriptase inhibitor; TDF, tenofovir disoproxil fumarate; QD, once daily.

^a^NRTIs were TDF/FTC or ABC/3TC.

**Supplementary Figure 2.** **Study design of DRIVE-SHIFT**


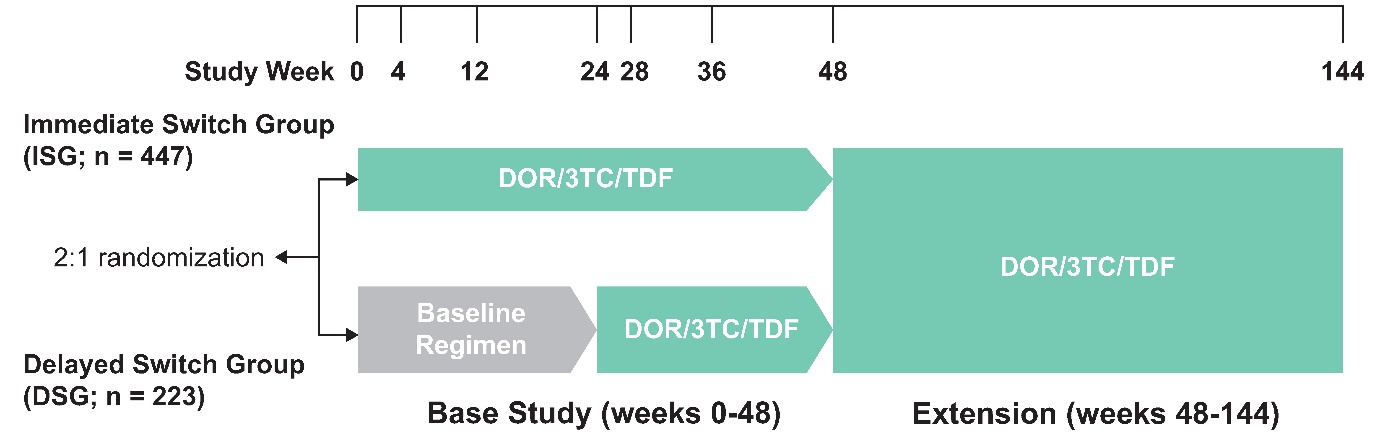


3TC, lamivudine; DOR, doravirine; DSG, delayed switch group; ISG, immediate switch group; TDF, tenofovir disoproxil fumarate.

**Supplementary Figure 3.** **Analysis of factors impacting the probability of (A) weight loss or (B) stable weight versus weight gain from week 96 to week 192, DOR continued group, DRIVE-FORWARD and DRIVE-AHEAD.**


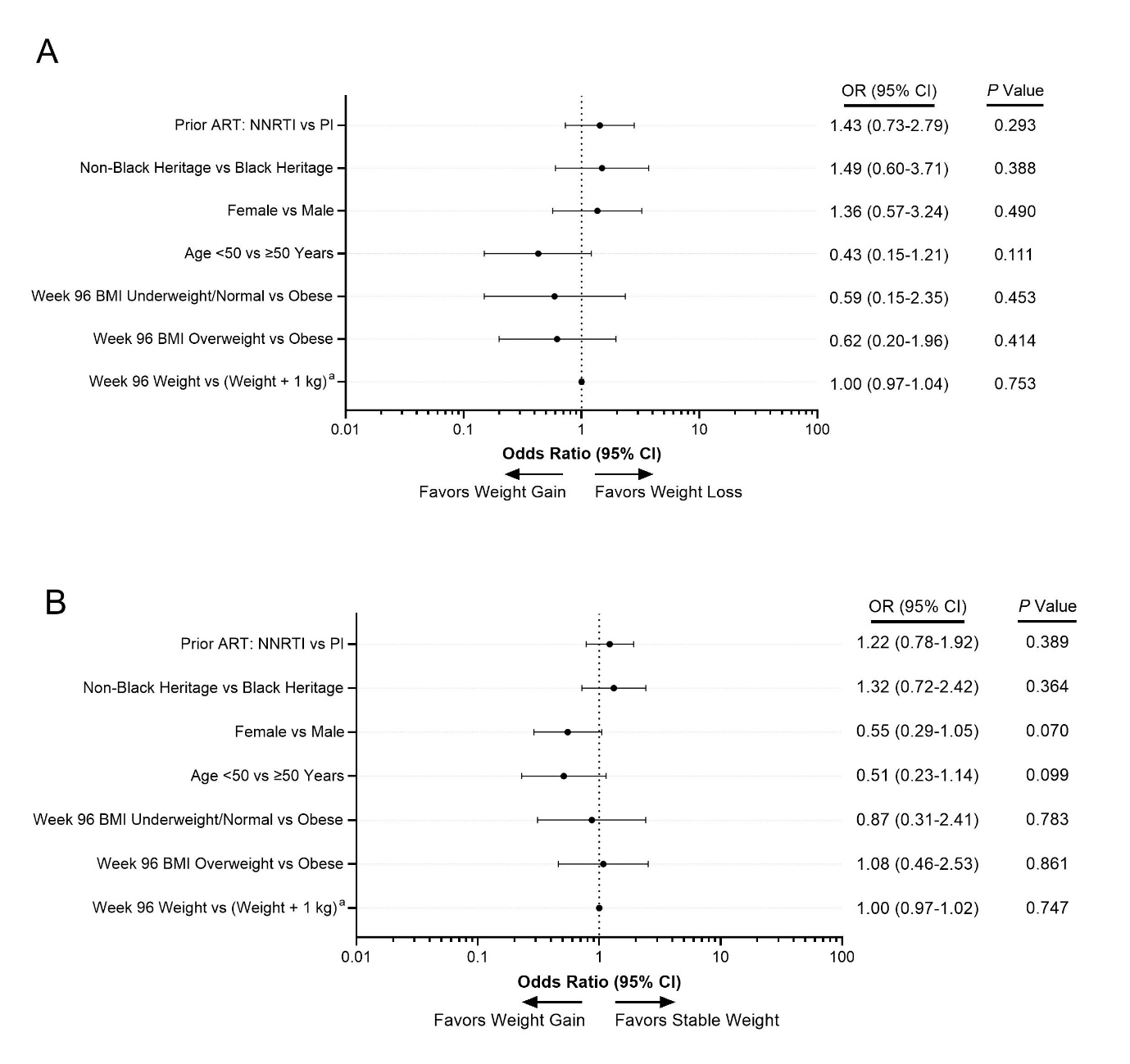


ART, antiretroviral therapy; BMI, body mass index; DOR, doravirine; NNRTI, non-nucleoside reverse transcriptase inhibitor; OR, odds ratio; PI, protease inhibitor.

^a^For each additional kg of weight, the odds ratio for week 96 weight versus (weight + 1 kg) represents the ratio of the odds of two groups, one with weight X and another with weight (X + 1).
